# Structure of mouse cytosolic sulfotransferase SULT2A8 provides insight into sulfonation of 7α-hydroxyl bile acids

**DOI:** 10.1016/j.jlr.2021.100074

**Published:** 2021-04-16

**Authors:** Kai Wang, Yan-Chun Chan, Pui-Kin So, Xing Liu, Lu Feng, Wing-Tai Cheung, Susanna Sau-Tuen Lee, Shannon Wing-Ngor Au

**Affiliations:** 1 Faculty of Science, School of Life Sciences, The Chinese University of Hong Kong, Shatin, Hong Kong; 2 University Research Facility in Life Sciences, The Hong Kong Polytechnic University, Hung Hom, Hong Kong; 3 Faculty of Medicine, School of Biomedical Sciences, The Chinese University of Hong Kong, Shatin, Hong Kong; 4 Center for Protein Science and Crystallography, School of Life Sciences, The Chinese University of Hong Kong, Shatin, Hong Kong

**Keywords:** bile acid metabolism, protein structure, homeostasis, liver, sulfotransferase, mSULT2A8, X-ray crystallography, 7-hydroxyl, sulfonation, CA, cholic acid, DHEA, dehydroepiandrosterone, GST, glutathione-*S*-transferase, LCA, lithocholic acid, Na C, sodium cholate, Na CDC, sodium chenodeoxycholate, Na T-C, sodium taurocholate, OH, hydroxyl, PAP, adenosine 3’,5’-diphosphate, PAPS, 3’-phosphoadenosine-5’-phosphosulfate, PB, 3’-phosphate binding, PSB, 5’-phosphate-sulfate binding, SULT, sulfotransferase, *T*_m_, transition midpoint

## Abstract

Cytosolic sulfotransferases (SULTs) catalyze the transfer of a sulfonate group from the cofactor 3’-phosphoadenosine 5’-phosphosulfate to a hydroxyl (OH) containing substrate and play a critical role in the homeostasis of endogenous compounds, including hormones, neurotransmitters, and bile acids. In human, SULT2A1 sulfonates the 3-OH of bile acids; however, bile acid metabolism in mouse is dependent on a 7α-OH sulfonating SULT2A8 via unknown molecular mechanisms. In this study, the crystal structure of SULT2A8 in complex with adenosine 3’,5’-diphosphate and cholic acid was resolved at a resolution of 2.5 Å. Structural comparison with human SULT2A1 reveals different conformations of substrate binding loops. In addition, SULT2A8 possesses a unique substrate binding mode that positions the target 7α-OH of the bile acid close to the catalytic site. Furthermore, mapping of the critical residues by mutagenesis and enzyme activity assays further highlighted the importance of Lys44 and His48 for enzyme catalysis and Glu237 in loop 3 on substrate binding and stabilization. In addition, limited proteolysis and thermal shift assays suggested that the cofactor and substrates have protective roles in stabilizing SULT2A8 protein. Together, the findings unveil the structural basis of bile acid sulfonation targeting 7α-OH and shed light on the functional diversity of bile acid metabolism across species.

Bile acids play an essential role in intestinal absorption of lipids and function as signaling molecules and activators of nuclear receptors that regulate lipid, cholesterol, glucose, energy, and bile acid metabolism (1, 2, 3, 4, 5, 6). However, bile acids possess detergent-like properties, and they disrupt plasma and mitochondrial membrane lipids at high concentration (7, 8). In human, excessive accumulation of bile acids can lead to pathologic-hepatobiliary changes like cholestasis (7). Bile acid elimination involves sulfonation, glucuronidation, and hydroxylation (9, 10). Sulfonation of bile acids abolishes their cholestatic activity by increasing their water solubility, limiting their enterohepatic recirculation, and enhancing their fecal excretion (11, 12, 13).

Cytosolic sulfotransferases (SULTs) are a large family of enzymes catalyzing the sulfonation of both xenobiotics and endogenous compounds, including hormones, neurotransmitters, and bile acids (14, 15, 16, 17). Sulfonation involves the transfer of a sulfonate (SO_3_^−^) group from the universal cofactor 3’-phosphoadenosine-5’-phosphosulfate (PAPS) to a hydroxyl (OH) group of the substrate to generate a sulfonated product (18, 19, 20). Based on their amino acid sequence similarities, mammalian cytosolic SULTs are classified into six families (SULT1-SULT6), of which SULT1 and SULT2 are the largest and most important families for xenobiotic and endobiotic metabolism (21). Protein sequence alignment of all these six SULT family members shows that the 5’-phosphate-sulfate binding (PSB) and 3’-phosphate binding (PB) motifs for cofactor binding are conserved. On the contrary, regions for substrate binding are highly divergent, reflecting the broad spectrum of substrates across SULT families (15, 22, 23, 24, 25, 26, 27). For example, members in SULT1 family are mainly responsible for the metabolism of phenols, estrogens, and catecholamines (28, 29, 30, 31), whereas SULT2 enzymes mainly catalyze hydroxysteroids and bile acids (32, 33, 34).

Members in the SULT2 family are further divided into SULT2A and SULT2B subfamilies. In human (h), hSULT2A1 is the sole isoform of SULT2A subfamily possessing a broad substrate reactivity toward numerous endogenous hydroxysteroids and bile acids (35, 36). hSULT2A1 detoxifies bile acids through monosulfonation of the 3-OH group in liver (32, 37). Although seven *Sult2a* genes (*mSult2a1*-*mSult2a7*) have been previously identified, their transcriptional expression is suppressed or silenced in adult mice except *mSult2a7* (38), and their biological functions are all unclear. Since mSULT2A1 shares high sequence identity with hSULT2A1, it was considered as an orthologue of 3-OH sulfonating hSULT2A1 and played an essential role in bile acid detoxification as in human. However, previous investigation of the bile acid pool in mouse shows that 7-OH bile acid-monosulfates were the predominant forms, and their levels are particularly higher in males than in females (35, 39, 40). This contradicts with the hepatic mRNA expression pattern of *Sult2a1*, which was exclusively detected in female mice (38), indicating that sulfonation of bile acids at 7-OH requires another SULT. Our team has recently identified SULT2A8 as the new member of SULT2A subfamily in mouse. SULT2A8 is predominantly expressed in male adults, and recombinant SULT2A8 preferentially catalyzes the sulfonation of 7α-OH bile acids including cholic acid (CA), chenodeoxycholic acid, and their conjugated forms (41). In addition, RNA-sequencing analysis shows that *Sult2a8* is abundantly expressed among all the eight *Sult2a* genes in mouse liver (42). Recent physiological studies suggested that SULT2A8-mediated bile acid sulfonation is involved in bile acid detoxification (43), and the downregulation of SULT2A8 expression is associated with the formation of cholesterol gallstone (44). Our *in* *vivo* study on a SULT2A8-haplodeficient mouse model also demonstrated that SULT2A8 is critical in maintaining the homeostasis of hepatic taurine-conjugated CA (45), a predominant proportion in mouse bile acid pool (46). Together, the pathophysiological role of SULT2A8 becomes clearer.

Accumulating studies have demonstrated the mechanism of 3-OH sulfonation (37, 47, 48, 49); however, the structural basis of bile acid sulfonation at 7-OH by SULT2A8 is less understood. Interestingly, multiple sequence alignment of all SULTs reveals that a conserved catalytic histidine (His99 in hSULT2A1) is substituted by a leucine in SULT2A8. Although it has been proposed that nonconserved His48 serves as the catalytic residue for the bile acid sulfonation in SULT2A8 (50), the underlying mechanistic features are not understood. In the present study, we have solved the crystal structure of mSULT2A8 in complex with a cofactor analogue adenosine 3’,5’-diphosphate (PAP) and a 7α-OH substrate CA. Structural comparison with the hSULT2A1 reveals that the unique 7α-OH sulfonation of bile acid in SULT2A8 is contributed by a differential mode of substrate binding and the nonconserved catalytic residue His48. Furthermore, the interaction between SULT2A8 and the substrate, especially the role of the substrate binding loops, has been explored.

## Materials and methods

### Cloning, protein expression, and purification

The truncated form of SULT2A8 encoding residues 4–282 was amplified from pRSET-SULT2A8 fusion plasmid previously described (41) using the forward primer 5’-CGCGGATCCGAATTTCTGTGGATAGAAGG-3’ and reverse primer 5’-CCGCTCGAGTTATTCCCATGAAAAGAGCT-3’. The PCR product was cloned into a pGEX-6P-3 vector (GE Healthcare) using BamHI and XhoI restriction sites for the expression of an N-terminal glutathione-*S*-transferase (GST)-tagged SULT2A8. This recombinant plasmid was also used for site-directed mutagenesis to generate various SULT2A8 mutants for biochemical assays as indicated in the text. All the plasmids were confirmed by Sanger sequencing and transformed into *Escherichia coli* BL21(DE3) for protein expression. Cells were grown in lysogeny broth medium at 37°C, and protein expression was induced with 0.2 mM isopropyl β-d-1-thiogalactopyranoside for 16 h at 20°C.

SULT2A8 was purified from inclusion bodies as previously described with slight modifications (51). Briefly, cells were resuspended in 50 mM Hepes, pH 8.0, 500 mM NaCl, and 20 mM β-mercaptoethanol, followed by adding 1 mg/ml lysozyme for cell lysis at 4°C for 30 min. Resuspension was incubated with 10 μg/ml DNase I at 4°C for 30 min and 1% *N*-lauroylsarcosine at room temperature for 30 min. Cell debris was pelleted by centrifugation at 20,000 *g* for 20 min prior to brief sonication. The supernatant was added with 2% Triton-X100 and 20 mM CHAPS before binding to Glutathione Sepharose 4B (GE Healthcare) at room temperature for 30 min. The beads were washed with the same buffer, and GST tag was cleaved with PreScission protease at 4°C for overnight. SULT2A8 protein was purified by a HiLoad 16/60 Superdex 75 prep grade column (GE Healthcare) with 20 mM Hepes, pH 8.0, 150 mM NaCl, and 4 mM 1,4-dithiothreitol. Fractions were pooled and concentrated for crystallization and activity assays.

### Crystallization and structure determination

SULT2A8 protein was cocrystallized with PAP and CA. Briefly, protein at a concentration of ∼8 mg/ml was incubated with 4 mM PAP and 4 mM CA on ice for 30 min, followed by 1:1 mixing with crystallization buffer. An initial crystal screening was performed with Index crystallization screen kit (Hampton Research). The initial hit (condition G10 in Index screen) was optimized with grid screening and additive (1,4-dioxane). Higher quality crystals were obtained by 2:1 mixing of ligands and 3% 1,4-dioxane-incubated SULT2A8 protein with reservoir buffer (0.1 M MES, pH 5.1, 21% polyethylene glycol 3350, and 0.2 M MgCl_2_). Crystals were grown with the sitting-drop vapor diffusion method at 16°C and cryoprotected by soaking in reservoir buffer containing 20% glycerol prior to flash freezing. Diffraction data were collected using in-house FR-E+ SuperBright generator (Rigaku) equipped with an R-AXIS IV++ IP detector (Rigaku). Data were processed and integrated using iMosflm (52). Phase determination was achieved by molecular replacement in Phenix (53), using the ligand-free hSULT2A1 (Protein Data Bank [PDB] ID: 3F3Y) as a searching model. mSULT2A8 model was built and refined with Coot (54) and Phenix (53), respectively. A twin law (h, -k, -l) was applied during the model refinement. Figures were generated with both PyMOL (Schrodinger) (55) and UCSF Chimera (56). The protein coordinates and structural factors were deposited in PDB under the accession code 7D1X (supplemental Data S1).

### SULT2A8 enzyme activity

The enzyme activities of SULT2A8 were determined by HPLC method through the quantification of PAP generated from the sulfonation of substrates as previously described (57). In brief, 100 μl of reaction mixture consisted of 100 mM sodium acetate buffer (pH 5.5) or sodium phosphate buffer (pH 7.5), 2.5 mM MgCl_2_, 15 μg purified protein, 100 μM substrate, and 50 μΜ PAPS as described (41). Control experiment without the substrate was performed in parallel. The reaction was started by adding the substrate to the reaction mixture and further incubated at 37°C for 1 h. The reaction was terminated by adding 100 μl of ice-cold methanol containing 50 μΜ of theophylline as an internal standard. The reaction mixture was then centrifuged at 14,000 *g* for 10 min under 4°C, and the supernatant was subjected to HPLC analysis. Substrate- and PAPS-free reaction mixtures containing 2, 5, 10, 20, and 50 μM of PAP were prepared in the same way for PAP calibration curve.

HPLC assays were performed with a 1260 Infinity II LC System equipped with a 1290 Infinity II Diode Array Detector (Agilent). Injection volume was 25 μl, and analytes were separated with methanol-water (20:80; v/v) containing 75 mM KH_2_PO_4_, 50 mM NH_4_Cl, and 1 mM 1-octylamine (pH 4.75) at a flow rate of 1.0 ml/min on a reverse phase XBridge BEH C18 column (130 Å; 5 μm, 4.6 mm × 250 mm) (Waters). The column temperature was 30°C, and peaks were detected at 254 nm wavelength (supplemental Fig. S1). The peak area ratios of PAP to internal standard were used to plot the calibration curve as well as calculate the generated PAP in each reaction sample. Specific activities were calculated as generated PAP (nanomoles) per minute per milligram of protein (58).

### Limited proteolysis

Limited proteolysis of SULT2A8 was performed in the presence/absence of 500 μM PAP and/or sodium chenodeoxycholate (Na CDC). In brief, 20 μg of purified SULT2A8 was incubated with different concentrations of trypsin (0, 50, 100, or 200 ng) in digestion buffer 90 mM Tris, pH 8.5, 2 mM CaCl_2_, and 4 mM 1,4-dithiothreitol. The reactions were adjusted to a final volume of 20 μl and incubated at room temperature for 30 min. Digestion was terminated with SDS-PAGE loading dye and heated. The digestion reactions were then analyzed by SDS-PAGE and Coomassie blue staining.

### Thermal shift assay

The effects of bile acids and steroids on SULT2A8 protein stability were examined by a fluorescent-based thermal shift assay (59). The protein sample was first incorporated with a hydrophobic fluorescent probe. The unfolding of the protein was then measured by monitoring the increase of the fluorescence signal during temperature increase. A fitting procedure allows the calculation of transition midpoint (*T*_m_). Briefly, 500 μg/ml of protein in 20 mM Hepes, pH 8.0, 150 mM NaCl, and 4 mM 1,4-dithiothreitol was incubated with 250 mM of PAP and/or tested compounds for 20 min on ice. This was followed by mixing 20 μl of prepared protein with 5 μl of 500-fold diluted JBS Thermofluor Dye (Jena Bioscience) in the same buffer. Control experiments were set using buffer and dye mixture without proteins. Sample mixtures were initially incubated at 4°C for 2 min, followed by heating in a ramp rate of 1°C/min starting from 4 to 95°C with a CFX96 Touch Real-Time PCR Detection System (Bio-Rad). The excitation wavelength was set at 483 nm, whereas emission wavelength was 568 nm. Nonlinear fit was solved, and *T*_m_ was calculated by a Boltzmann sigmoidal equation with XLfit (IDBS).

### Statistical analysis

Biochemical results are expressed as mean ± standard deviation, and differences were compared with the unpaired Student's *t*-test using GraphPad Prism (GraphPad Software). *P* < 0.05 is considered as statistically significant, and *P* values were indicated categorically: ∗*P* < 0.05, ∗∗*P* < 0.01, ∗∗∗*P* < 0.001, and ∗∗∗∗*P* < 0.0001.

## Results

### Overall structure of SULT2A8

In our earlier efforts, full-length His-tagged SULT2A8 was cocrystallized with PAP or both PAP and Na CDC; however, the crystals were only diffracted to resolutions of 3.6 and 7.9 Å, respectively. A new SULT2A8 expression plasmid with a cleavable GST fusion tag was therefore constructed. This new SULT2A8 construct that has the first three residues from the N terminus removed, when cocrystallized with both PAP and CA, resulted in needle-like crystals. Grid screening on pH produced cube-shaped crystals from pH 4.9 to 5.8. A 2.5 Å complete dataset was collected from a crystal obtained under pH 5.1 (supplemental Table S1). SULT2A8 was crystallized in space group P4_2_ with two copies of SULT2A8 per asymmetric unit and Matthews coefficient of 2.51 Å^3^Da^−1^. Analysis of the integrated data with Xtriage in Phenix suggested a possibility of twinning with twinning fraction of 0.44 (L-test with <|*L*|> = 0.42, <*L*^2^> = 0.24) and a twin law (h, -k, -l). After phase determination by molecular replacement, this twin law was applied in the refinement. The model of SULT2A8 was finally refined to *R*_work_/*R*_free_ value of 23.85%/27.15% with both PAP and CA successfully built in each chain. The two chains of SULT2A8 are almost identical with root mean square deviation of Cα atoms of 0.486. There is no interpretable electron density for residues 227–231 in chain A and residues 90–93, 128–141, 229–238, and 277–280 in chain B, indicating that these regions are flexible. The structural analysis reported here is mainly based on the more complete chain A model.

**Fig. 1:**
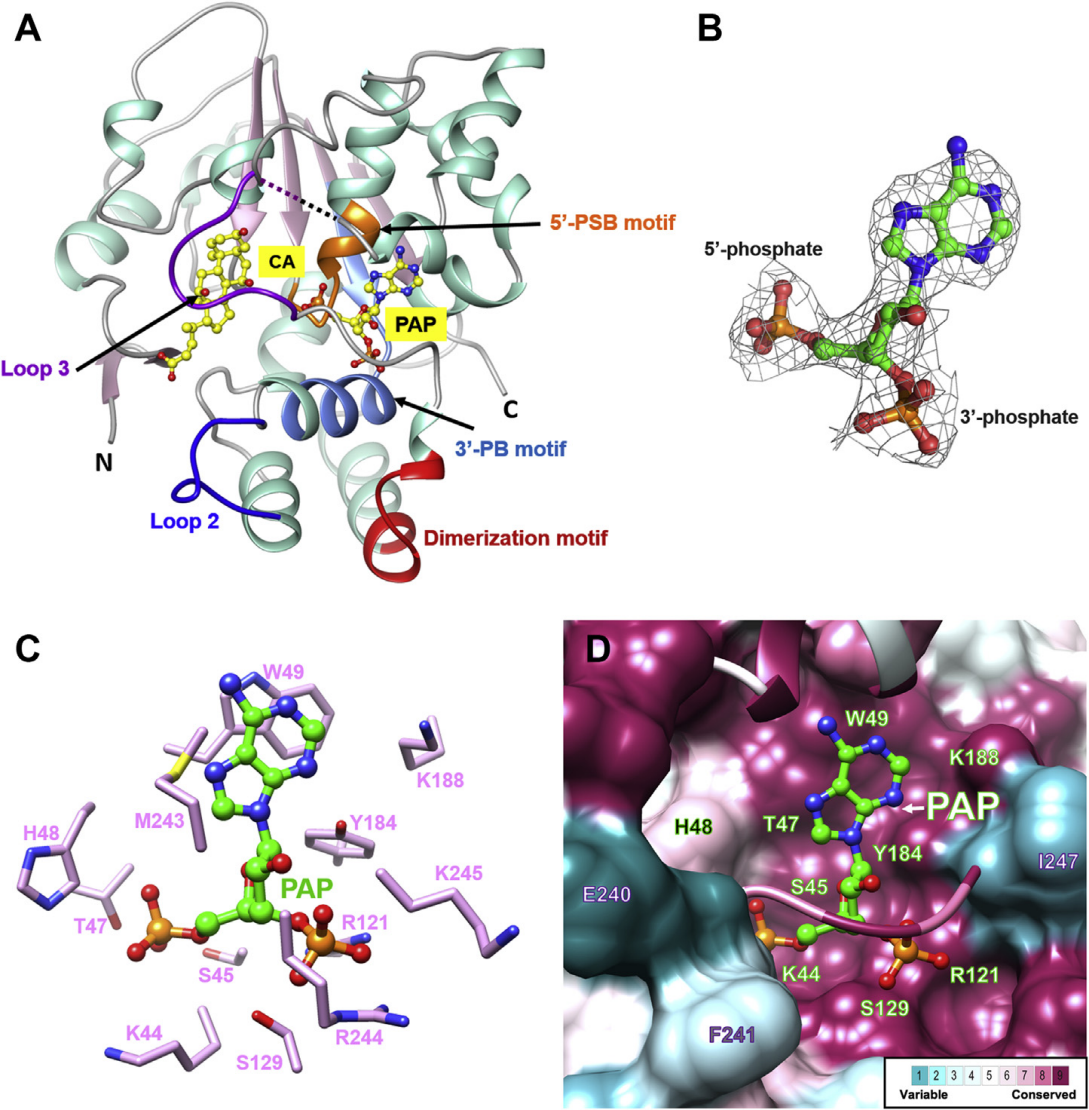
Overall structure of SULT2A8. A: SULT2A8 model is presented in cartoon mode. Conserved 5’-PSB, 3’-PB, and dimerization motifs are indicated. Substrate binding loops 2 (blue) and 3 (purple) of SULT2A8 are shown, and the missing region in loop 3 including residues 227–231 is marked as dashed line. The bound cofactor PAP and substrate CA are shown in yellow ball-and-stick mode. B: PAP in the SULT2A8 model and its 2*F*o-*F*c electron density map at contour level of 1.0 σ. C: The PAP binding pocket. Residues involved in the PAP binding are shown as sticks. D: Conservation of amino acids among all mouse SULT2A isoforms (mSULT2A1-mSULT2A8) was analyzed via ConSurf (60) and mapped to the SULT2A8 structure. Residues 220–225 and 242–245 are shown as cartoon to aid the visualization of the PAP binding pocket.

The overall structure of SULT2A8 is a classical SULT α/β fold comprised of a central four-stranded parallel β-sheet flanked by 14 α-helixes (Fig. 1A). SULT2A8 possesses a conserved dimerization motif near the C terminus, and analysis of the crystal packing revealed dimerization of SULT2A8 resembled that of hSULT2A1. The structure also showed an extra electron density in the PAPS binding pocket (Fig. 1B). A PAP molecule was modeled so that its 5’- and 3’-phosphate groups interact with the 5’-PSB and 3’-PB motifs in the PAPS binding pocket, respectively (Fig. 1A). Apart from a His48, all residues lining in the mSULT2A8 PAP pocket are highly conserved with hSULT2A1 and the other mouse SULT2A isoforms (mSULT2A1-mSULT2A7), which include Lys44, Ser45, Thr47, and Trp49 in 5’-PSB motif, Arg121 and Ser129 in 3’-PB motif, Tyr184 and Lys188 in helix α9, and Met243, Arg244, and Lys245 in the loop for PAPS binding, indicating that both human and mouse SULT2A members share a common binding mode for the cofactor (Fig. 1C, D).

**Fig. 2:**
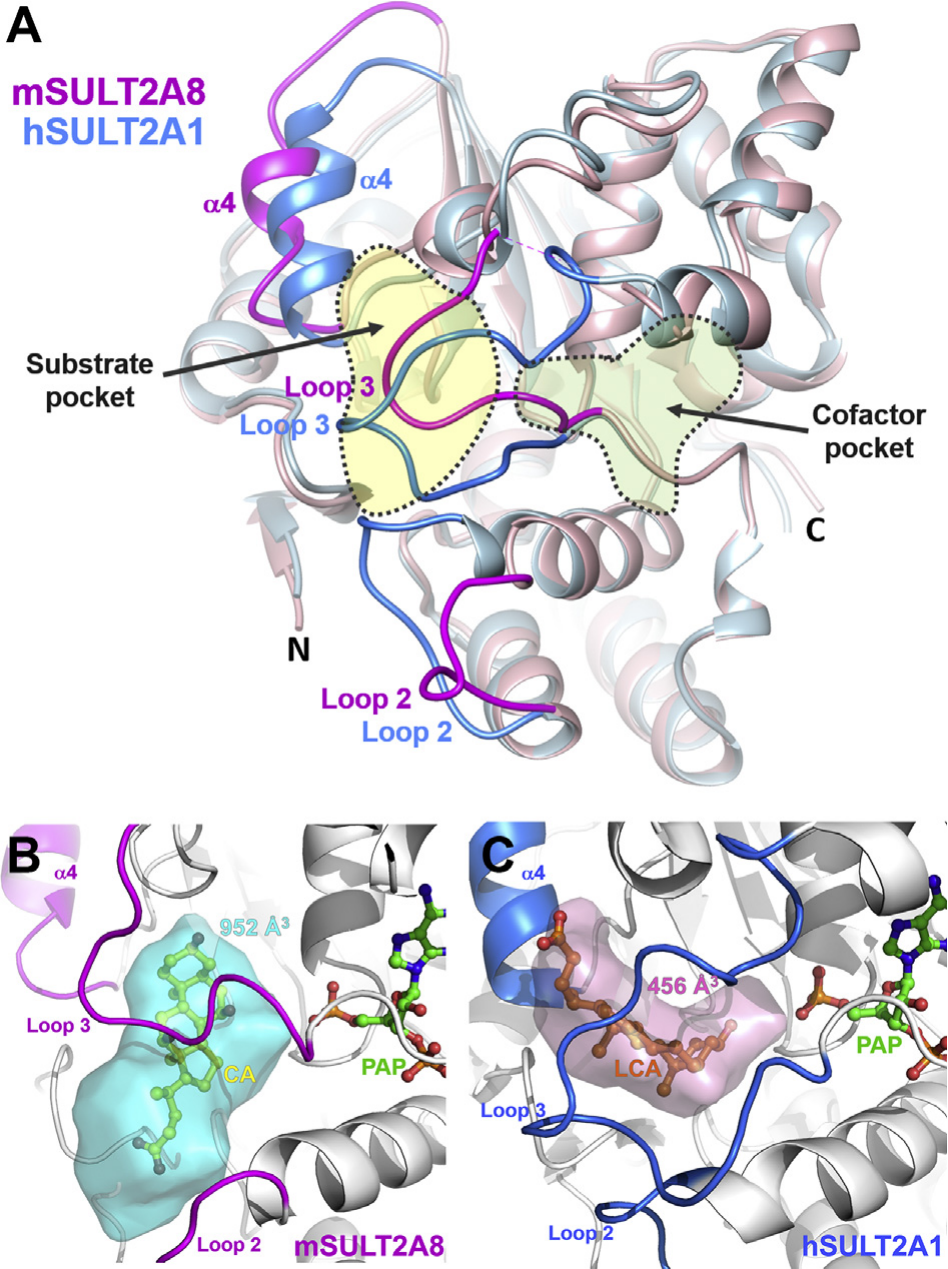
Comparison of the overall structure and substrate cavity of mSULT2A8 with hSULT2A1. A: Structural superimposition of mSULT2A8 (pink) with hSULT2A1 (blue) (PDB ID: 3F3Y) was performed via MatchMaker in UCSF Chimera (56). B and C: Substrate binding cavities of mSULT2A8 (B) and hSULT2A1 (C) were generated by POCASA program (62) using the substrate-free models. Substrate cavity volume was calculated by UCSF Chimera (56).

Structural homology search by DALI (61) reveals that mSULT2A8 shares the highest structural similarity (Z-score 35.5) with hSULT2A1 (PDB ID: 3F3Y) (37) across all the available protein structure coordinates. The two structures are well superimposed except for the loop 2 and loop 3 regions for substrate binding (Fig. 2A). In mSULT2A8, its loop 2 is in a more “opened” conformation, whereas its loop 3 (three residues shorter than the respective region in hSULT2A1) shows a tighter substrate encapsulation. The amino acid sequences of loops 2 and 3 in mSULT2A8 are hypervariable when compared with all SULT2A members in human and mouse (supplemental Fig. S2). In addition, a ∼3.5 Å outward shift of helix α4 and its flanking loops (residues 80–92) in mSULT2A8 was noticed when compared with hSULT2A1. All these structural differences contribute to a ∼2-fold-broader substrate cavity in mSULT2A8 (952 Å^3^) (Fig. 2B) compared with hSULT2A1 (456 Å^3^) (Fig. 2C).

### SULT2A8 adopts a unique substrate recognition mode

**Fig. 3:**
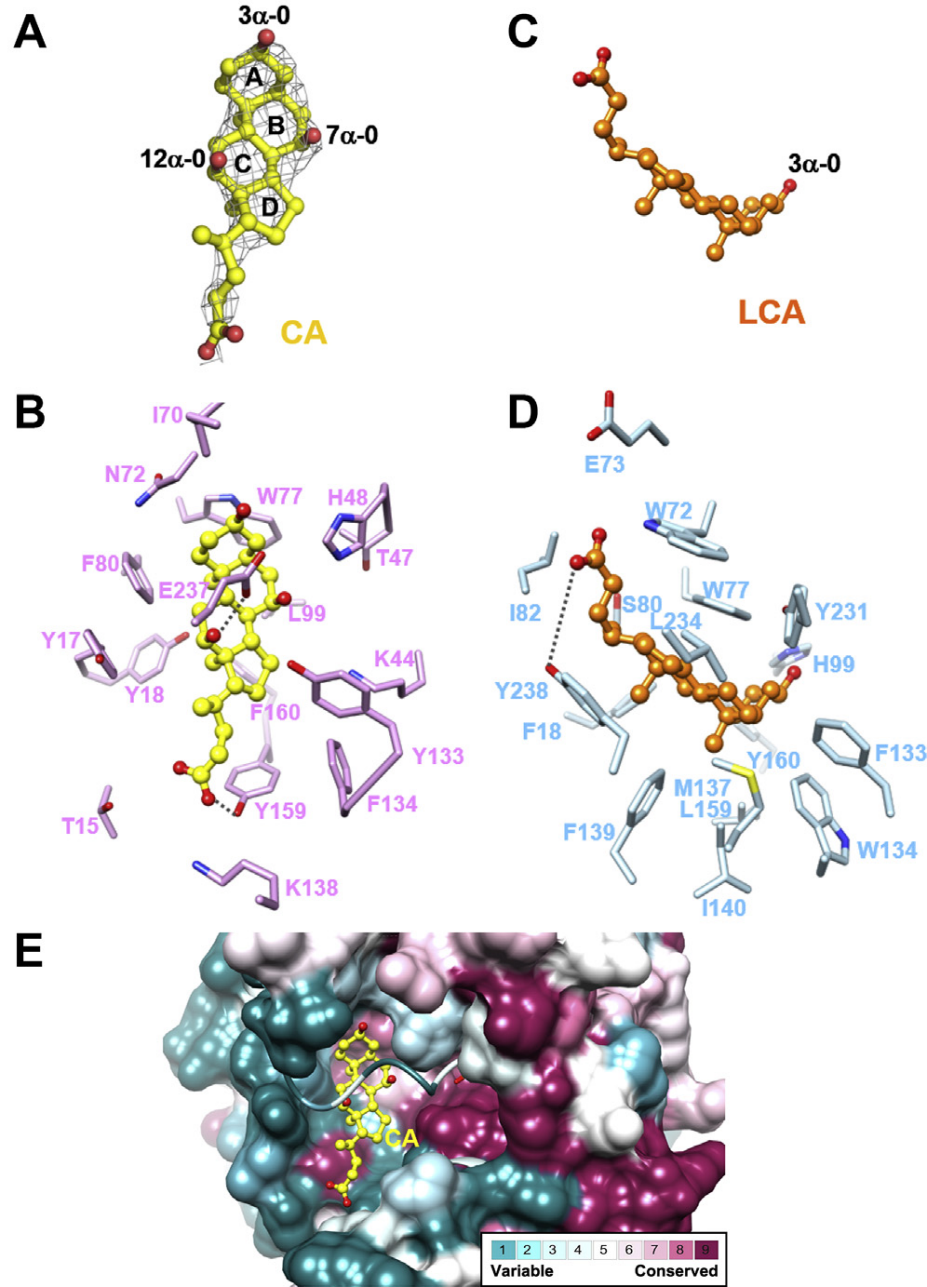
Distinct binding mode of bile acid in mSULT2A8 and hSULT2A1. A: CA in mSULT2A8 model and its 2*F*o-*F*c electron density map at contour level of 1.0 σ. A and C: Both CA (A) and LCA (C) are composed of a four-ring steroid backbone (rings A, B, C, and D) forming a “chair” conformation and a 5-carbon side chain terminating in a carboxylic acid. CA carries two extra OH groups (7α-OH and 12α-OH) on the steroid ring when compared with LCA. B and D: Residues in the substrate binding pockets of mSULT2A8 (B) and hSULT2A1 (PDB ID: 3F3Y) (D) are shown as sticks. E: Conservation of amino acid sequences among all mouse SULT2A isoforms (mSULT2A1-mSULT2A8) was analyzed via ConSurf (60). Molecular surface of substrate binding pocket in mSULT2A8 is colored based on the ConSurf conservation score. Residues 232–244 in the substrate binding loop 3 are shown as cartoon to aid the visualization of the PAP binding pocket.

In the present study, SULT2A8 was cocrystallized with PAP and its native substrate CA to understand its mechanistic features for substrate recognition and sulfonation. The 2*Fo-Fc* electron density map for the substrate CA was clear that the 3α-, 7α-, and 12α-OH ends of CA could be well modeled into the map (Fig. 3A). The overall hydrophobic nature of the substrate pocket is contributed by Tyr17, Tyr18, Ile70, Trp77, Phe80, Leu99, Tyr133, Phe134, Tyr159, and Phe160 (Fig. 3B). Specifically, Trp77, Phe80, and Leu99 interact with the hydrophobic side of CA backbone. In the hydrophilic side of CA (3α-, 7α-, 12α-OH, and its side chain), orientation of the substrate in the binding pocket is further enhanced by hydrogen bonds between Oε of Glu237 and 12α-O of CA (2.7 Å), as well as side chain OH of Tyr159 (Oη) and CA molecule (24-O) (2.3 Å).

Structural comparison of the substrate binding pockets between mSULT2A8 and hSULT2A1 complexed with PAP and lithocholic acid (LCA), a bile acid lacking both 7α- and 12α-OH groups (Fig. 3C, D), further showed that mSULT2A8 adopts distinct characteristics for bile acid recognition. Noteworthy, CA in mSULT2A8 is orientated with all three OH groups exposed toward the solvent environment and rotated at about 90° when compared with LCA in hSULT2A1. The unique orientations of the two substrates are likely contributed by various hydrophobic residues in the binding pocket. Although Trp77 is conserved between mSULT2A8 and hSULT2A1, its indole ring stacks with steroid ring A of CA in mSULT2A8 instead of rings B-D of LCA in hSULT2A1. The hydrophobic contacts of the bile acid backbones are also facilitated by the nonconserved residues: Phe80 in mSULT2A8 and Tyr238 in hSULT2A1. Furthermore, the orientation of the substrate appears to be directed by the distinct set of hydrogen bond interaction with the carboxylic side chains of the substrates. Tyr159 in loop between helixes α7 and α8 of mSULT2A8 and Tyr238 in loop 3 of hSULT2A1 form hydrogen bonds with the carboxylic side chains of the respective substrates. The conservation of the substrate binding pocket across the eight SULT2A isoforms in mouse was also analyzed and mapped to the mSULT2A8 structure (Fig. 3E). The results suggest that mSULT2A8 would have distinct substrate binding property from the other mSULT2A isoforms.

### Mechanism of 7α-OH sulfonation in SULT2A8

**Fig. 4:**
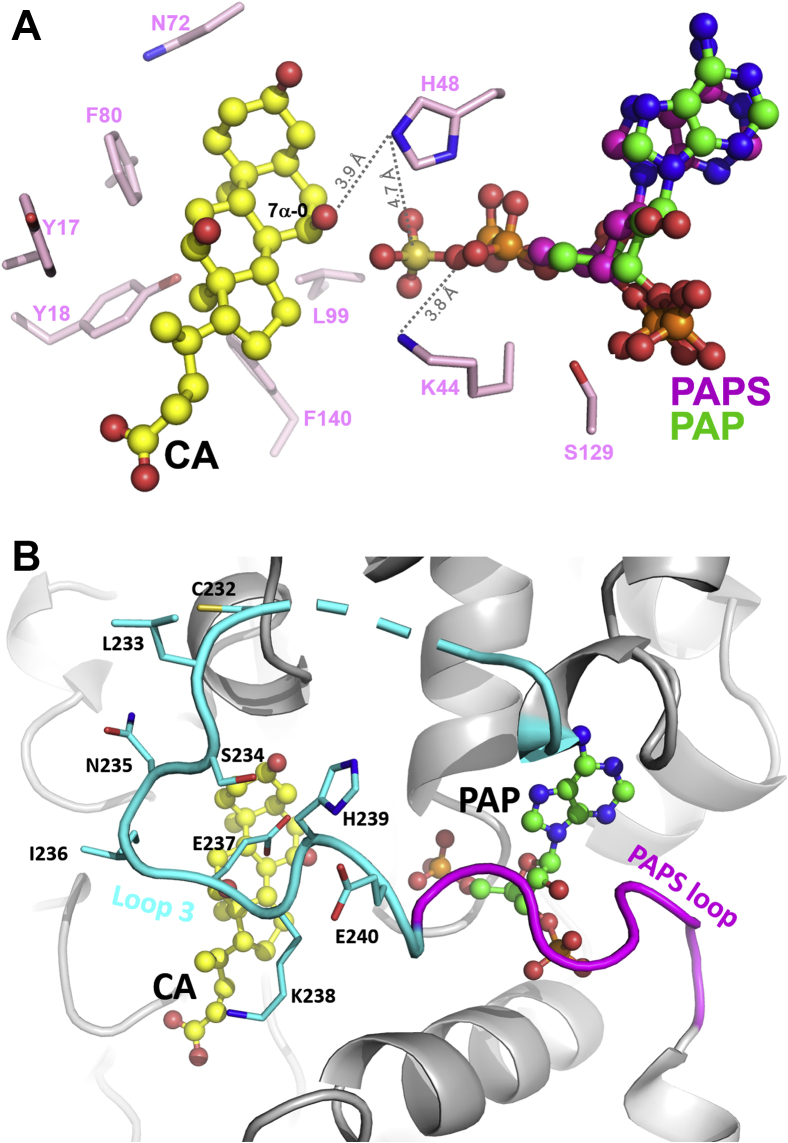
Active site and catalytic residues of SULT2A8. A: Structure of hSULT2A1 (PDB ID: 4IFB) was aligned with mSULT2A8 to simulate the position of the transferring sulfonate group of PAPS. Distances between His48 and CA 7α-O, His48 and PAPS sulfonate group, Lys44 and bridging oxygen in PAPS are shown as dashed lines. B: Substrate binding loop 3 (cyan) adjacent to the loop for PAPS binding (magenta) embraces the two ligand pockets of SULT2A8. Residues in loop 3 are shown as sticks. Region (residues 227–231) with missing electron density is shown in dashed line.

Previous biochemical and structural studies demonstrated that the catalytic reaction of SULTs requires three conserved residues, His99, Lys44, and Ser129 (in hSULT2A1 sequence). The conventionally accepted mechanism is that residue His99 acts as a catalytic base for the deprotonation of the target OH of the substrate with subsequent nucleophilic attack of the sulfur atom of PAPS (63). The hydrolysis of the sulfonate-PAP bond is mediated by Lys44 whose position is modulated by Ser129, as demonstrated in human SULT1E1 (64). While both Lys44 and Ser129 are conserved in mSULT2A8, His99 is substituted by Leu99, which is on the hydrophobic face of the substrate CA (Fig. 3B), indicating that mSULT2A8 employs an alternative residue as a catalytic base for the sulfonation reaction. To simulate the position of the sulfonate group in the cofactor and its transfer to the 7α-OH of CA, mSULT2A8 structure was superimposed with a hSULT2A1 model cocrystallized with PAPS (PDB ID: 4IFB) (Fig. 4A). Of interest, His48 lining on the 5’-PSB motif of mSULT2A8 is in close proximity to both 7α-OH of CA (3.9 Å) and sulfonate group of PAPS (4.7 Å). The side chain Nε atom of Lys44 is in a distance of 3.8 Å to the PAP/sulfonate bridging oxygen. Residue Ser129 on helix α6 is positioned between Lys44 and the 3’-phosphate group of PAP. Together, it appears that mSULT2A8 adopts catalytic triad His48, Lys44, and Ser129 for sulfonation.

**Fig. 5:**
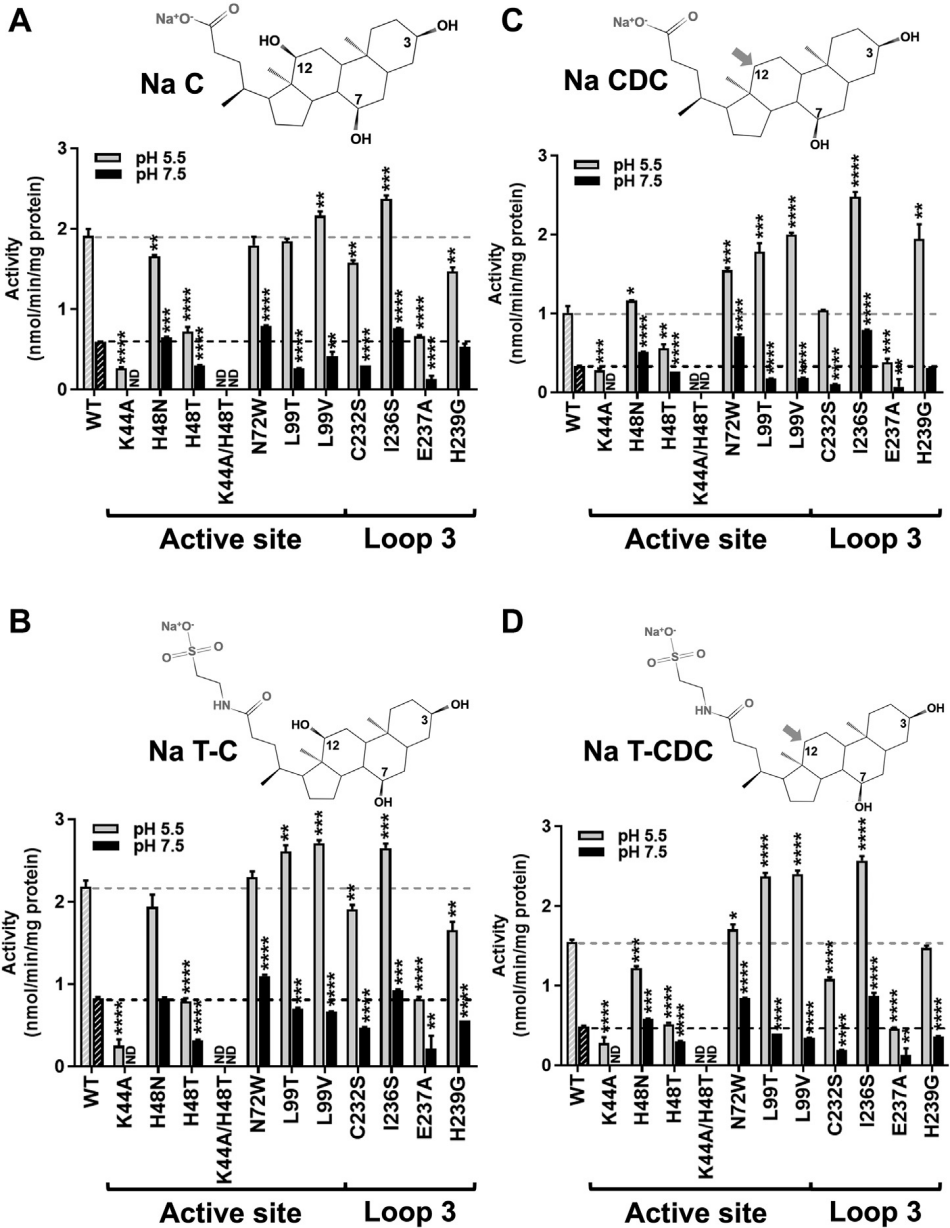
Enzyme activity of SULT2A8 and its mutants toward different bile acids. Sulfonation activities of SULT2A8 toward Na C (A), Na T-C (B), Na CDC (C), and sodium taurochenodeoxycholate (Na T-CDC) (D) under both pH 5.5 and 7.5 are determined and compared with corresponding WT activities (dashed lines). Results are expressed as mean and standard deviation (n = 3), and differences of each mutant were compared with WT using unpaired Student's *t*-test. *P* < 0.05 is considered as statistically significant, and *P* values were indicated categorically: ∗*P* < 0.05, ∗∗*P* < 0.01, ∗∗∗*P* < 0.001, and ∗∗∗∗*P* < 0.0001. The two-dimensional structures of substrates are shown in each figure. Na C (A) and Na T-C (B) possess 3α-, 7α-, and 12α-OH groups, whereas Na CDC (C) and Na T-CDC (D) lack the 12α-OH group (gray arrows). Side chains of Na T-C and Na T-CDC are conjugated with taurine compared with their nonconjugated forms.

The importance of His48 in mSULT2A8 activity has previously been studied, that is, substitutions of His48 with asparagine and threonine led to 10% residual enzyme activity and nondetectable activity, respectively (50). Here, we carried out a thorough mutagenesis analysis to validate the functional roles of the residues in the active site and on loop 3 as revealed from the crystal structure of SULT2A8. Specifically, SULT2A8 mutants K44A, H48N, H48T, N72W, L99T, L99V, C232S, I236S, E237A, and H239G were created. The enzyme activities of SULT2A8 WT and mutants toward various types of bile acids and steroids were compared under both optimized (pH 5.5) (41) and physiological (pH 7.5) pH. For 7α-OH-lacking substrates like LCA, androsterone, dehydroepiandrosterone (DHEA), and pregnanolone, sulfonation activity was not detected in all SULT2A8 constructs. In line with our previous study (41), SULT2A8 WT and its mutants showed higher sulfonation activities toward 7α-OH bile acids in acidic condition (Fig. 5) (supplemental Tables S2 and S3). Sulfonation activities of mutant H48N toward sodium cholate (Na C), sodium taurocholate (Na T-C), Na CDC, and sodium taurochenodeoxycholate were comparable to the WT enzyme. However, activities of mutant H48T toward these four substrates were decreased by ∼50% or more under both pH conditions. The overall effects of these two substitution mutants are comparable with the study of Shimohira *et al.* (50), although the extent of activity reduction is less dramatic in our study. The discrepancy could likely be due to the differences in assay buffer condition. Interestingly, a significant decrease of enzyme activity was observed in mutant K44A, which resulted in 70–90% loss of enzyme activity under pH 5.5 and nondetectable activity under pH 7.5. The results of K44A and H48T mutants were further validated by using LC/MS to quantitate the generated CA-monosulfate (supplemental Fig. S3). Mutations of both Lys44 and His48 (K44A/H48T) led to complete loss of sulfonation activity toward all tested substrates, suggesting that these two residues, especially Lys44, are critical for 7α-OH sulfonation of bile acids.

We further explored the roles of the two nonconserved residues Asn72 and Leu99 in the active site of mSULT2A8, by making substitution with their equivalent residue in hSULT2A1 based on the sequence alignment (supplemental Fig. S2). As shown in Fig. 5, mutant N72W showed some enhancement of enzymatic activity, in particular for substrate Na CDC. As revealed from the crystal structure, Glu237 forms a hydrogen bond with 12α-OH of CA. We speculate that the absence of 12α-OH in Na CDC may allow some flexibility to orientate the bile acid, and mutation of Asn72 to a bulky tryptophan may turn Na CDC toward His48 for sulfonation. Surprisingly, this mutant displayed low activity (0.32 nmol/min/mg protein) to a sole 3α-OH containing androsterone but not LCA (supplemental Table S2). Androsterone differs from LCA by the absence of carboxylic acid side chain at 5-C position. This result suggests that while Trp72 in mutant N72W modulates the orientation of 3α-OH of the substrates, the carboxylate side chain of LCA is hydrogen bonded with Tyr159 and therefore limits its movement to His48 for catalysis. For residue Leu99 in mSULT2A8, its equivalent substitute in most SULTs is the conserved catalytic histidine. Here, we constructed mutants L99V and L99T to investigate if hydrophobic property of the Leu99 side chain is necessary. Both mutants L99V and L99T hindered the bile acid sulfonation under pH 7.5; however, their activities toward most bile acids increased under pH 5.5. These findings further support that Leu99 is involved in SULT2A8 sulfonation, probably by stabilizing the bound substrate through hydrophobic contact. Yet, we cannot explain the pH-dependent differences in sulfonation activities in these two Leu99 mutants.

Loop 3 is the largest but divergent substrate binding loop in SULTs, which may have a role in substrate specificity and thus sulfonation efficiency (65, 66). Sequence alignment shows that the loop 3 of SULT2A8 possesses more hydrophilic residues than the other human and mouse SULT2A members (supplemental Fig. S2). This hydrophilic nature may promote the binding of the substrates and their orientation to expose the hydrophilic plane to the solvent environment. To validate this, SULT activities of mutants on Cys232, Ile236, Glu237, and His239 located on the loop 3 (Fig. 4B) were examined. As shown in Fig. 5, Ile236 is the only hydrophobic residue in the loop 3 closing to the bound CA. Interestingly, mutating Ile236 to a hydrophilic serine significantly enhanced the sulfonation of all the 7α-OH bile acids tested, suggesting that hydrophilicity of loop 3 assists 7α-OH sulfonation in mSULT2A8. Our hypothesis was further supported by mutant E237A, which lost over 50% activities to all the substrates. Cys232 and His239 are unique residues in SULT2A8 among mouse and human SULT2A subfamily. Compared with WT, significant decreases of enzyme activities toward 7α-OH bile acids were detected when mutating Cys232 or His239 to the conserved serine or glycine, respectively. A 2-fold increase of H239G sulfonation activity on Na CDC lacking a 12α-OH was observed only under pH 5.5, however with unknown reason.

### Cofactor and substrates enhance SULT2A8 structural stability

**Fig. 6:**
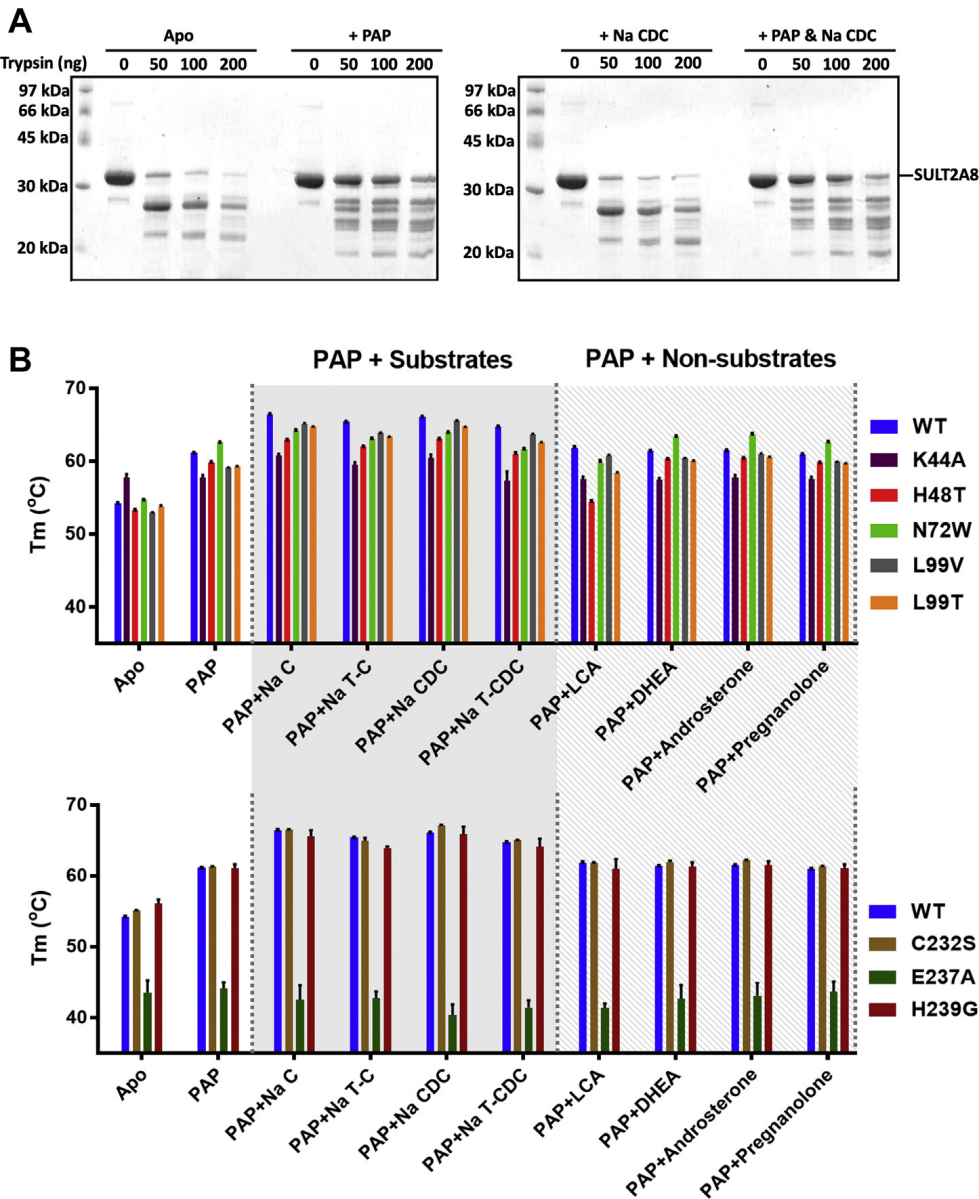
SULT2A8 stability in the presence of cofactor and substrates. A: Trypsin limited proteolysis study. Purified SULT2A8 was coincubated with or without PAP and Na CDC as indicated in the trypsin digestion reactions. Digested proteins were electrophoresed by SDS-PAGE and detected by Coomassie blue staining. B: SULT2A8 WT and its mutants with substitution in the active site (upper panel) or loop 3 (lower panel) were coincubated with PAP, native/non-native substrates as indicated. The reaction was assayed using JBS Thermofluor Dye system. Fluorescent at 568 nm was detected with excitation wavelength of 483 nm under heating in a ramp rate of 1°C/min. *T*_m_ was calculated by solving the nonlinear fit through a Boltzmann sigmoidal equation and presented in means and standard deviation (n = 3). Differences of each ligand-treated protein were compared with its corresponding apo form using unpaired Student’s *t*-test, and significance was indicated in the text.

Crystal structures of SULT are currently limited to the ligand-bound forms, suggesting that binding of cofactor and/or substrates is required for protein stability and compact structure formation (26, 67). Here, we carried out limited proteolysis to characterize the effects of ligands on SULT2A8 stability (Fig. 6A). SULT2A8 protein was cleaved into two major fragments upon trypsin digestion in the absence of PAP and Na CDC; however, the addition of PAP notably prevented SULT2A8 from limited proteolysis. In the presence of Na CDC, the proteolytic pattern is similar to that of the apo form. The addition of both PAP and Na CDC did not further protect SULT2A8 from trypsin digestion. These results indicate that SULT2A8 stability is largely enhanced by the cofactor.

To further explore the roles of cofactor and substrates on SULT2A8 stability, thermal stability shift assays were carried out (Fig. 6B) (supplemental Table S4). Addition of PAP in the reaction mixture caused a pronounced shift in the melting transition of the SULT2A8 WT protein and an increase of *T*_m_ from 54 to 61°C. The *T*_m_ values were further increased when both PAP and native substrates (Na C, Na T-C, Na CDC, or sodium taurochenodeoxycholate) were added. However, incubating SULT2A8 with both PAP and nonsubstrates, such as LCA, DHEA, androsterone, or pregnanolone, showed comparable thermal denaturation profile to that of PAP only. Compared with apo WT protein, all *T*_m_ shifts were statistically significant. These results suggest that both cofactor and substrate protect SULT2A8 from unfolding.

Thermal denaturation transition profiles of mutants K44A, H48T, N72W, L99V, and L99T were also obtained. Similar to the WT enzyme, the *T*_m_ values of these mutants were significantly increased when PAP was included. When both PAP and native substrates were present, further increase of *T*_m_ was observed in mutants H48T, L99V, and L99T. These thermal stability profiles are similar to those of the WT enzyme. Remarkably, the presence of PAP did not enhance the thermal stability in mutant K44A, further confirming that conserved Lys44 is critical for cofactor binding. Interestingly, although the *T*_m_ value of N72W was increased when PAP was added, no further changes in *T*_m_ were detected upon coincubation with substrates (native or non-native). For mutations on loop 3, mutants C232S and H239G in apo form exhibited similar *T*_m_ as that of WT, whereas mutant E237A showed a drastic reduction of *T*_m_. This result suggests that Glu237 plays a critical role in stabilizing SULT2A8 structure. In addition, incubating E237A with native or non-native substrates failed to improve the protein stability. These results further indicate that interaction of the enzyme with bile acids is mediated by Glu237 on loop 3.

## Discussion

Excessive accumulation of bile acids can lead to hepatobiliary change like cholestasis (68). SULT-mediated sulfonation is an important pathway to maintain the bile acid homeostasis (69). Unlike 3α-OH sulfonation of bile acids through SULT2A1 in human, 7-OH sulfonation is the pathway for bile acid detoxification in mouse (40). Compared with 3-sulfates, 7-sulfates are more resistant to hydrolysis and bacterial metabolism in gut (70), which reduce their intestinal reabsorption (71). As a 7α-OH bile acid-preferred SULT, SULT2A8 appears to be the major enzyme for bile acid elimination in mouse (72). Interestingly, a conserved catalytic histidine (His99 in hSULT2A1) is substituted by a leucine in SULT2A8, suggesting that SULT2A8 adopts a distinct catalytic mechanism. Here, the crystal structure of SULT2A8 in complex with PAP and CA has revealed its structural basis for the substrate recognition and 7α-OH sulfonation. While SULT2A8 shares conserved binding mode for the cofactor as in other SULTs, structural comparisons with SULTs complexed with steroid-like substrates, including hSULT2A1, hSULT2B1, hSULT1A1, hSULT1E1, and mSULT1E1, indicate that the substrate in mSULT2A8 is oriented entirely different. Unlike the sulfonate-accepting OH of the substrates is in close proximity to the conserved catalytic histidine (His99 in hSULT2A1), the 7α-OH of CA positions toward both His48 and Lys44 as well as the 5’-phosphate group of cofactor in mSULT2A8 (supplemental Fig. S4). Our model provides structural evidence for how His48 acts as a catalytic residue for the deprotonation of the substrate as proposed by the previous study (50). Results from our site-directed mutagenesis studies further imply that both Lys44 and His48 are key residues for 7α-OH sulfonation, although only 40% enzyme activity was retained when either residue was mutated. Similar to hSULT2A1 (36), pH 5.5 is the optimized pH of mSULT2A8 enzyme activity. With reference to the theoretical p*K*_*R*_ value of histidine (p*K*_*R*_ = 6.0), His48 would be in a protonated state at pH 5.5. This would then disagree with the role of His48 as a base to deprotonate 7α-OH of bile acids. However, the p*K*_*R*_ value of His48 in apo SULT2A8 model calculated with PROPKA (73) program is 5.0, suggesting the surrounding residues and microenvironment in the active site of SULT2A8 lower its p*K*_*R*_. Noteworthy, p*K*_*R*_ of His48 in both PAPS- and CA-bound forms was reduced to 3.1 and 3.6, respectively, and further to 2.6 when both ligands are present. This finding is comparable to the p*K*_*R*_ 2.5 of His99 calculated from the hSULT2A1 model (PDB ID: 3F3Y), indicating that the binding of cofactor and substrate modulate the actual p*K*_*R*_ of catalytic histidine for the deprotonation of bile acids.

Furthermore, mSULT2A8 structure is featured with unique conformations of loop 2 and loop 3. Compared with the other human SULT1 and SULT2 isoforms cocrystallized with both cofactor and substrates, loop 2 and loop 3 in mSULT2A8 are in a “relaxed” and “tighter” conformation, respectively (supplemental Fig. S5). The loops 2 and 3 in mSULT2A8 appear to stabilize the substrates by interacting with their side chain and steroid backbone, respectively. It has been proposed that, in loop 3, residues Tyr238 of hSULT2A1, Phe247 of hSULT1A1, Leu247 of hSULT1A3, and Leu249 of hSULT2B1_v1 interact and prevent the release of bound substrate (49). In mSULT2A8, equivalent residue Glu237 forms a hydrogen bond with 12α-OH of CA (3α-OH, 7α-OH, and 12α-OH). The importance of Glu237 in loop 3 is further supported by the enzyme activity and thermal stability assays that significant loss of sulfonation activity and thermal stability were found in E237A mutant. It also explains why lower sulfonation activity of mSULT2A8 toward the Na CDC (3α-OH and 7α-OH) lacking a 12α-OH was observed when compared with Na C. In addition, the enzyme activity data suggest that the adjacent residue Ile236 also plays a role in substrate specificity. The sulfonation activities of I236S mutant toward four tested 7α-OH bile acids increased to comparable levels. Ile236 is highly conserved among human and mouse SULT2A isoforms (isoleucine, valine, or alanine) (supplemental Fig. S2). From the hSULT2A1 structure (PDB ID: 3F3Y), this hydrophobic residue locates at the hinge of loop 3 and may be involved in maintaining the conformation of the loop. However, specifically how Ile236 in loop 3 modulates the substrate specificity is unclear. In our study, higher SULT2A8 activities toward taurine-conjugated bile acids compared with their nonconjugated forms were observed. We speculate that the relaxed conformation of loop 2 in SULT2A8 may facilitate the recognition of the side chain-conjugated bile acids. To test this, molecular docking of T-CA to SULT2A8 structure was performed by AutoDock Vina (74). While the backbone of T-CA overall aligned well with CA molecule, binding of substrate could be stabilized by hydrogen bonds between the taurine oxygen of T-CA and Nζ of both Lys138 (3.3 Å) and Lys238 (3.1 and 3.3 Å) in loops 2 and 3, respectively (supplemental Fig. S6). This clue is in line with the essential role of SULT2A8 in the elimination of T-CA, the most predominant bile acid in mouse (46, 75).

In addition, our limited proteolysis and thermal shift assays suggest the role of cofactor in stabilizing SULT2A8 protein. The presence of native substrates further enhanced the SULT2A8 thermal stability, which is in good agreement with molecular dynamic simulation on hSULT2A1 (76). This result indicates that the substrate binding may induce more compact structural fold through hydrogen bonds and hydrophobic interactions within the active site (77). It also explains why crystallization of SULT2A8 in the absence of either cofactor or substrate could not be obtained. In mouse, the hepatic levels of PAPS (∼20 μM) (78) and T-CA (140 μM) (46) are much higher than their *K*m of SULT2A8, 2.9 μM (79) and 21.1 μM (50), respectively. We speculate that most SULT2A8 protein is stabilized by both PAPS and T-CA in hepatocytes, that the high sulfonation efficiency and homeostasis of T-CA can be probably maintained in mouse (45).

The present study demonstrates, for the first time, the mechanistic details of bile acid 7α-OH sulfonation mediated by His48 in mSULT2A8. The substrate recognition mode revealed from our structural model further provides insights to understand the sulfonation properties of the other SULT2A isoforms. Phylogram generated by multiple sequence alignment shows that the genetic distance of hSULT2A1 to mSULT2A1-mSULT2A7 is closer than to mSULT2A8. Their phylogenetic relationship can be demonstrated when we compare the binding of bile acid in hSULT2A1 and mSULT2A8 (Fig. 3B, D). Similar residues involved in the extensive hydrophobic contact with the bile acid backbone in hSULT2A1, including Phe139, Tyr231, and Leu234, are found in mSULT2A1-mSULT2A7 sequences. Contact of bile acid backbone in mSULT2A8 is mainly contributed by Phe80, Leu99, and Phe160. However, the former two residues are substituted by polar residues (threonine, serine, or asparagine) and a catalytic histidine, respectively, in hSULT2A1 and mSULT2A1-mSULT2A7 (supplemental Fig. S2). We speculate that these seven mSULT2A isoforms would share similar substrate recognition and catalytic property with hSULT2A1. Interestingly, mSULT2A3 possesses an extra histidine at position 48 apart from the conserved His99. When CA was docked to an mSULT2A3 homology model generated from SWISS-MODEL (80), its orientation is comparable to that of LCA in hSULT2A1. The distance between His99 and 3α-OH (3.6 Å), and between His48 and 7α-OH (5.0 Å), of CA in the docking model suggests that mSULT2A3 may possess a dual sulfonation property preferably targeting 3α-OH.

In human, as the sole SULT2A isoform, hSULT2A1 preferentially catalyzes an androgen precursor DHEA to maintain androgen homeostasis and LCA to decrease its cytotoxicity. Like human, mSULT2A1 also catalyzes both DHEA and LCA in liver (81, 82). Enhanced sulfonation through upregulation of mSULT2A1, mSULT2A2, mSULT2A4, and mSULT2A6 expression, especially mSULT2A1, is a major route for LCA elimination under hypercholanemia caused by the deficiency of Na T-C cotransporting polypeptide, a hepatic transporter of conjugated bile acids (83). However, diminished expression of mSULT2A8 and PAPS synthase 2 increases the level of hydrophobic primary bile acids and results in the formation of cholesterol gallstone in hypothyroid mice (44). Together with the differential hepatic expression of SULT2A members in adult mouse (42), these findings imply their specific roles in bile acid metabolism. Inducible expression of mSULT2A1, mSULT2A2, mSULT2A4, and mSULT2A6 is essential for the detoxification of LCA, which is a minor but very toxic secondary bile acid, whereas high and constant expression of mSULT2A8 helps maintain a proper level of primary bile acids, which contribute the major proportion of total bile acid pool. To further elucidate the metabolic difference in bile acid sulfonation between human and mouse, future studies can be focused on structures and functions of the other mSULT2A isoforms.

## Data availability

The authors confirm that the data supporting the findings of this study are available within the article and its supplemental data. Protein structure (PDB ID: 7D1X) deposited in Worldwide PDB (http://www.wwpdb.org/) with a generated validation report on the structure quality will be released upon the acceptance of the present article.

## Conflict of interest

The authors declare that they have no known competing financial interests or personal relationships that could have appeared to influence the work reported in this article.
