# Supplementary material for: Structure of mouse cytosolic sulfotransferase SULT2A8 provides insight into sulfonation of 7α-hydroxyl bile acids
Source: J Lipid Res. 2021 Apr 16;62:100074. doi: 10.1016/j.jlr.2021.100074 (PMC8134075; doi:10.1016/j.jlr.2021.100074)
Supplement: Supplemental Figures S1–S6 and Tables S1–S4 [file mmc1.docx]

**SUPPLEMENTAL INFORMATION:**

**Structure of mouse cytosolic sulfotransferase SULT2A8 provides insight into sulfonation of 7α-hydroxyl bile acids**

Kai Wang^1^*, Yan-Chun Chan^1^, Pui-Kin So^3^, Xing Liu^1^, Lu Feng^1^, Wing-Tai Cheung^4^, Susanna Sau-Tuen Lee^1^, Shannon Wing-Ngor Au^1,2^ *

^1^School of Life Sciences, Faculty of Science, The Chinese University of Hong Kong, Shatin, Hong Kong.

^2^Center for Protein Science and Crystallography, School of Life Sciences, The Chinese University of Hong Kong, Shatin, Hong Kong.

^3^Department of Applied Biology and Chemical Technology, The Hong Kong Polytechnic University, Hung Hom, Hong Kong.

^4^School of Biomedical Sciences, Faculty of Medicine, The Chinese University of Hong Kong, Shatin, Hong Kong.

*****Co-corresponding authors

**Supplemental Table S1. Statistics of crystallographic data collection and refinement**

| **Data collection** | |
| --- | --- |
| Space group | P4_2_ |
| Unit cell dimensions |  |
| *a*, *b*, *c* (Å) | 93.9, 93.9, 71.3 |
| *α*, *β*, *γ* (°) | 90.0, 90.0, 90.0 |
| Resolution (Å) | 29.68-2.50 (2.64-2.50) |
| No. of molecules per asymmetric unit | 2 |
| Matthews coefficient (VM, Å^3^Da^-1^) | 2.51 |
| Solvent content (%) | 51.00 |
| No. of observations | 74809 (10757) |
| No. of unique reflections | 21605 (3135) |
| R_merge_ (%) | 0.157 (0.471) |
| *I/σI* ^1^ | 5.6 (2.2) |
| CC1/2 | 0.97 (0.73) |
| Completeness (%) | 99.0 (100.0) |
| Multiplicity | 3.5 (3.4) |
| **Refinement** | |
| R*_work_*/R*_free_* (%)^2^ | 23.85/27.15 |
| B factor | 55.0 |
| Clashscore | 8 |
| No. of atoms refined |  |
| Protein | 4316 |
| Ligands | 112 |
| Water | 84 |
| RMSDs^3^ |  |
| Bond lengths (Å) | 0.26 |
| Bond angles (°) | 0.47 |
| Ramachandran statistics (%) |  |
| Favored regions | 94.32 |
| Allowed regions | 5.68 |
| Outlier regions | 0 |

Values for the outer shell are shown in parentheses.

^1^*I/σI* was calculated from average < merged< Intensity >/standard deviation (< Intensity>) > from SCALA in CCP4 suite.

^2^R*_work_* = ∑(|F_obs_-F_calc_|/∑F_obs_), where F_obs_ = observed structure factor amplitude, and F_calc_ = structure factor calculated from model. R*_free_* is computed in the same manner as R*_work_*, but from a test set containing 5% of data excluded from the refinement calculation.

^3^RMSD, root-mean-square deviation.

**Supplemental Table S2. Enzyme activity of SULT2A8 mutants on bile acids and steroids under pH 5.5**

|  | **Activity (pmol/min/mg protein)** | | | | | | | |
| --- | --- | --- | --- | --- | --- | --- | --- | --- |
| **Substrates** | **Na C** | **Na T-C** | **Na CDC** | **Na T-CDC** | **LCA** | **Androsterone** | **DHEA** | **Pregnanolone** |
| OH position | 3α, 7α, 12α | 3α, 7α, 12α | 3α, 7α | 3α, 7α | 3α | 3α | 3β | 3β |
| WT | 1916.4 ± 79.6 | 2186.1 ± 72.7 | 1012.1 ± 86.4 | 1553.6 ± 27.0 | ND | ND | ND | ND |
| K44A | 267.7 ± 13.4^****^ | 257.4 ± 74.4^****^ | 280.7 ± 28.1^***^ | 280.3 ± 73.8^****^ | ND | ND | ND | ND |
| H48N | 1659.4 ± 20.2^**^ | 1945.7 ± 147.4 | 1166.4 ± 5.4^*^ | 1223.0 ± 25.9^***^ | ND | ND | ND | ND |
| H48T | 719.7 ± 62.1^****^ | 792.3 ± 42.1^****^ | 566.0 ± 48.0^**^ | 519.7.0 ± 16.3^****^ | ND | ND | ND | ND |
| K44A/H48T | ND | ND | ND | ND | ND | ND | ND | ND |
| N72W | 1796.4 ± 103.4 | 2300.4 ± 68.7 | 1548.2 ± 31.9^**^^*^ | 1710.8 ± 62.2^*^ | ND | 316.9 ± 3.4^***^ | ND | ND |
| L99T | 1844.7 ± 35.1 | 2613.2 ± 74.4^**^ | 1785.1 ± 111.4^***^ | 2378.0 ± 40.0^****^ | ND | ND | ND | ND |
| L99V | 2165.9 ± 49.7^**^ | 2718.5 ± 35.6^***^ | 2002.4 ± 28.3^****^ | 2401.8 ± 42.9^****^ | ND | ND | ND | ND |
| C232S | 1577.5 ± 30.2^**^ | 1911.4 ± 50.9^**^ | 1045.1 ± 8.7 | 1086.6 ± 24.8^****^ | ND | ND | ND | ND |
| I236S | 2375.0 ± 41.9^***^ | 2653.9 ± 52.6^***^ | 2479.2 ± 65.3^****^ | 2573.2 ± 58.4^****^ | ND | ND | ND | ND |
| E237A | 664.5 ± 10.6^****^ | 825.6 ± 25.8^****^ | 387.0 ± 45.8^***^ | 465.5 ± 16.0^****^ | ND | ND | ND | ND |
| H239G | 1743.0 ± 52.0^**^ | 1661.7 ± 96.8^**^ | 1945.1 ± 192.6^**^ | 1482.0 ± 26.2 | ND | ND | ND | ND |

Enzyme activity is calculated as generated PAP. Mean and SD (n=3) are presented and statistical difference between SULT2A8 WT and each mutant was determined by unpaired Student’s *t*-test with GraphPad Prism. ^*^, *P* < 0.05; ^**^, *P* < 0.01; ^***^, *P* < 0.001 and ^****^, *P* < 0.0001. ND, not detected.

**Supplemental Table S3. Enzyme activity of SULT2A8 mutants on bile acids and steroids under pH 7.5**

|  | **Activity (pmol/min/mg protein)** | | | | | | | |
| --- | --- | --- | --- | --- | --- | --- | --- | --- |
| **Substrates** | **Na C** | **Na T-C** | **Na CDC** | **Na T-CDC** | **LCA** | **Androsterone** | **DHEA** | **Pregnanolone** |
| OH position | 3α, 7α, 12α | 3α, 7α, 12α | 3α, 7α | 3α, 7α | 3α | 3α | 3β | 3β |
| WT | 597.4 ± 6.5 | 828.9 ± 17.4 | 339.4 ± 6.5 | 491.3 ± 7.8 | ND | ND | ND | ND |
| K44A | ND | ND | ND | ND | ND | ND | ND | ND |
| H48N | 652.9 ± 2.2^***^ | 827.9 ± 9.1 | 513.8 ± 3.8^****^ | 586.6 ± 8.8^***^ | ND | ND | ND | ND |
| H48T | 301.4 ± 5.2^****^ | 324.4 ± 1.3^****^ | 267.0 ± 0.9^****^ | 302.8 ± 8.4^****^ | ND | ND | ND | ND |
| K44A/H48T | ND | ND | ND | ND | ND | ND | ND | ND |
| N72W | 794.8 ± 3.2^****^ | 1100.8 ± 17.8^****^ | 708.9 ± 23.7^****^ | 844.8 ± 6.0^****^ | ND | ND | ND | ND |
| L99T | 264.7 ± 2.5^****^ | 704.9 ± 11.6^***^ | 171.8 ± 7.2^****^ | 399.7 ± 4.5^****^ | ND | ND | ND | ND |
| L99V | 418.9 ± 52.7^**^ | 665.7 ± 2.3^****^ | 184.0 ± 4.6^****^ | 346.5 ± 10.2^****^ | ND | ND | ND | ND |
| C232S | 300.9 ± 1.4^****^ | 476.3 ± 4.8^****^ | 101.8 ± 5.2^****^ | 194.8 ± 2.2^****^ | ND | ND | ND | ND |
| I236S | 768.9 ± 5.0^****^ | 932.1 ± 10.2^***^ | 799.2 ± 4.1^****^ | 875.8 ± 39.3^****^ | ND | ND | ND | ND |
| E237A | 135.3 ± 42.3^****^ | 221.9 ± 159.1^**^ | 72.7 ± 99.9^**^ | 128.4 ± 84.0^**^ | ND | ND | ND | ND |
| H239G | 533.3 ± 40.8 | 557.6 ± 0.8^****^ | 315.8 ± 1.8 | 372.9 ± 0.8^****^ | ND | ND | ND | ND |

Enzyme activity is calculated as generated PAP. Mean and SD (n=3) are presented and statistical difference between SULT2A8 WT and each mutant was determined by unpaired Student’s *t*-test with GraphPad Prism. ^*^, *P* < 0.05; ^**^, *P* < 0.01; ^***^, *P* < 0.001 and ^****^, *P* < 0.0001. ND, not detected.

**Supplemental Table S4. Tm of SULT2A8 mutants in presence of PAP and substrates**

|  |  |  | **Tm (^o^C)** | | | | | | | |
| --- | --- | --- | --- | --- | --- | --- | --- | --- | --- | --- |
| **Ligands** | **Apo** | **PAP** | **PAP**  **Na C** | **PAP**  **Na T-C** | **PAP**  **Na CDC** | **PAP**  **Na T-CDC** | **PAP**  **LCA** | **PAP**  **DHEA** | **PAP**  **Androsterone** | **PAP**  **Pregnanolone** |
| WT | 54.1±0.2 | 61.1±0.0 | 66.4±0.0 | 65.4±0.0 | 66.1±0.1 | 64.7±0.0 | 61.8±0.2 | 61.4±0.0 | 61.5±0.0 | 60.9 ± 0.1 |
| K44A | 57.8±0.5 | 57.7±0.4^NS^ | 60.7±0.3 | 59.5±0.3 | 60.4±0.5 | 57.3±1.3^NS^ | 57.5±0.3^NS^ | 57.4±0.3^NS^ | 57.7±0.4^NS^ | 57.5±0.3^NS^ |
| H48T | 53.2±0.1 | 59.8±0.0 | 62.9±0.0 | 62.0±0.0 | 63.0±0.0 | 61.0±0.0 | 54.4±0.1 | 60.3±0.0 | 60.4±0.0 | 59.8±0.0 |
| N72W | 54.7±0.0 | 62.6±0.0 | 64.3±0.0 | 63.0±0.1 | 64.0±0.0 | 61.6±0.1 | 59.8±0.3 | 63.4±0.1 | 63.6±0.2 | 62.6±0.0 |
| L99T | 53.8±0.1 | 59.3±0.0 | 64.7±0.0 | 63.3±0.0 | 64.7±0.0 | 62.5±0.1 | 58.3±0.2 | 60.0±0.0 | 60.6±0.0 | 59.7±0.0 |
| L99V | 53.0±0.0 | 59.1±0.0 | 65.2±0.0 | 63.9±0.0 | 65.5±0.0 | 63.7±0.0 | 60.7±0.1 | 60.4±0.0 | 61.0±0.1 | 59.9±0.0 |
| C232S | 55.1±0.0 | 61.3 ±0.0 | 66.5±0.1 | 64.9±0.4 | 67.1±0.1 | 65.0±0.1 | 61.8±0.1 | 61.9±0.2 | 62.2±0.0 | 61.3 ±0.1 |
| E237A | 43.5±1.7 | 44.1±0.8^NS^ | 42.5±2.0^NS^ | 42.7±1.0^NS^ | 40.4±1.5^NS^ | 41.4±1.0^NS^ | 41.4±0.6^NS^ | 42.7±1.9^NS^ | 43.1±1.8 | 43.7 ±1.4^NS^ |
| H239G | 56.1±0.6 | 61.1±0.5 | 65.5±0. 9 | 63.9±0.2 | 65.8±1.1 | 64.1±1.2 | 60.9±1.4 | 61.3±0.6 | 61.5±0.5 | 61.1 ±0.6 |

Mean and SD (n=3) are presented and statistical difference between SULT2A8 apo form and in presence of ligands were determined by unpaired Student’s *t*-test with GraphPad Prism. Compared to apo, all Tm shifts were statistically significant (*P* < 0.05) except data indicated with not significant (NS).


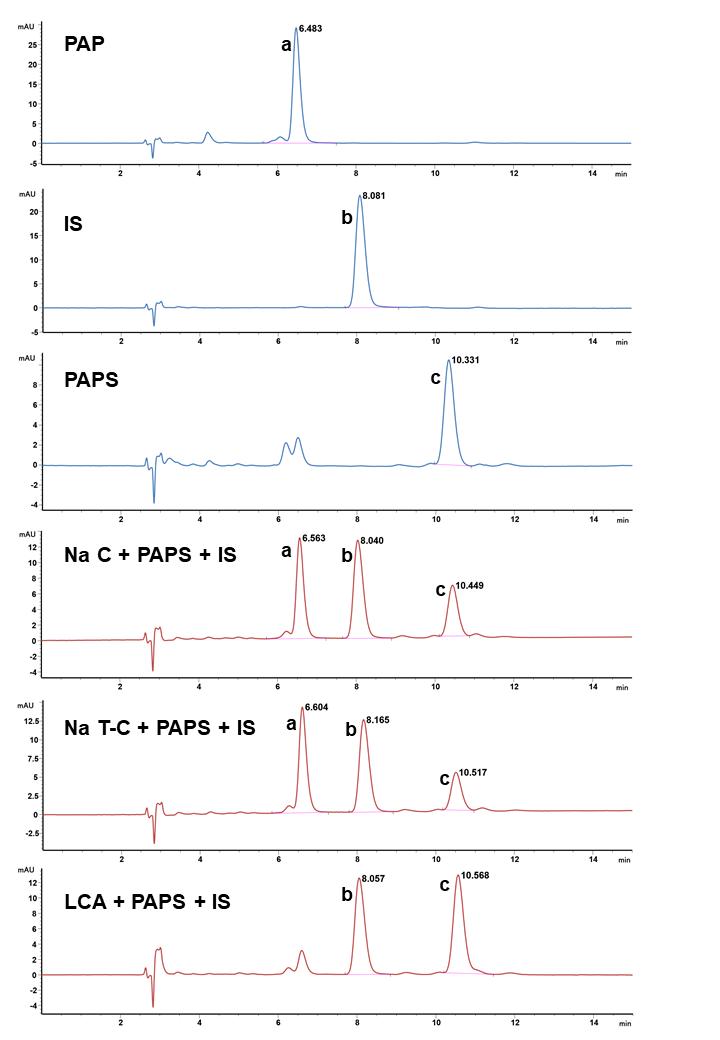


**Supplemental Fig. S1. Representative HPLC elution profiles of PAP, PAPS, and internal standard (IS).** Elution profiles of no-substrate reaction mixtures spiked with 100 μM of PAP (PAP), PAPS (PAPS), or internal standard (IS, theophylline) are shown. Representative elution of reaction mixtures of SULT2A8 WT towards Na C, Na T-C, and LCA (substrates + PAPS + IS) are compared in parallel. Retention time of PAP (a), IS (b), and PAPS (c) peaks are around 6.5, 8.1, and 10.5 min, respectively.


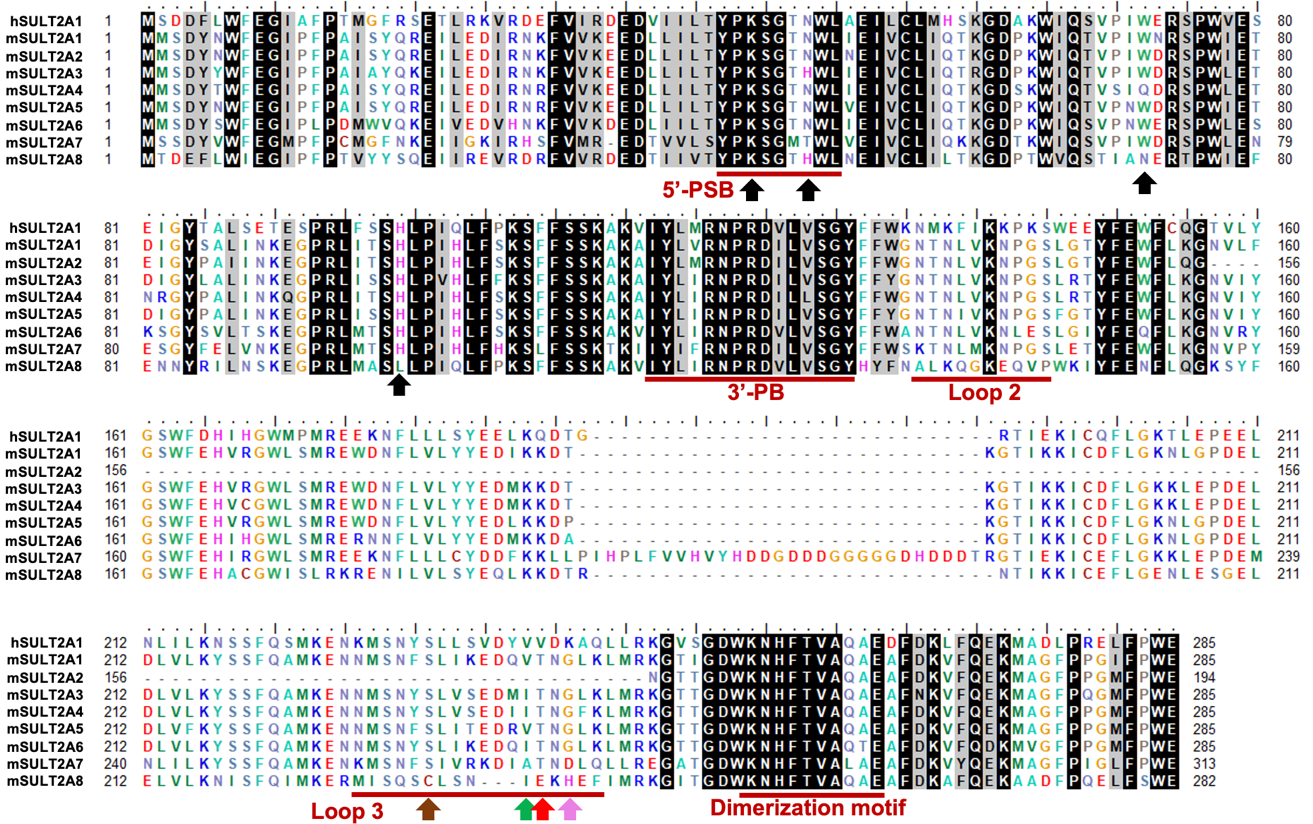


**Supplemental Fig. S2. Amino acid sequence alignment of human and mouse SULT2A subfamily isoforms.** Amino acid sequences of mSULT2A8 was aligned with mSULT2A1 (NP_001104766.1), mSULT2A2 (NP_033312.2), mSULT2A3 (NP_001095056.2), mSULT2A4 (NP_001095004.1), mSULT2A5 (NP_001171909.1), mSULT2A6 (NP_001074794.1), mSULT2A7 (NP_001171910.2) and hSULT2A1 (NP_003158.2) by ClustalW (84). Similar and identical residues among SULT2A isoforms are shaded in grey and black, respectively. Highly conserved 5’-PSB, 3’-PB and dimerization motifs are indicated. Non-conserved substrate binding loops 2 and 3 are shown. Residues in the active site and loop 3 used for site-direct mutagenesis are indicated with black and colored arrows (Cys232, brown; Ile236, green; Glu237, red; His239, pink) arrows, respectively.


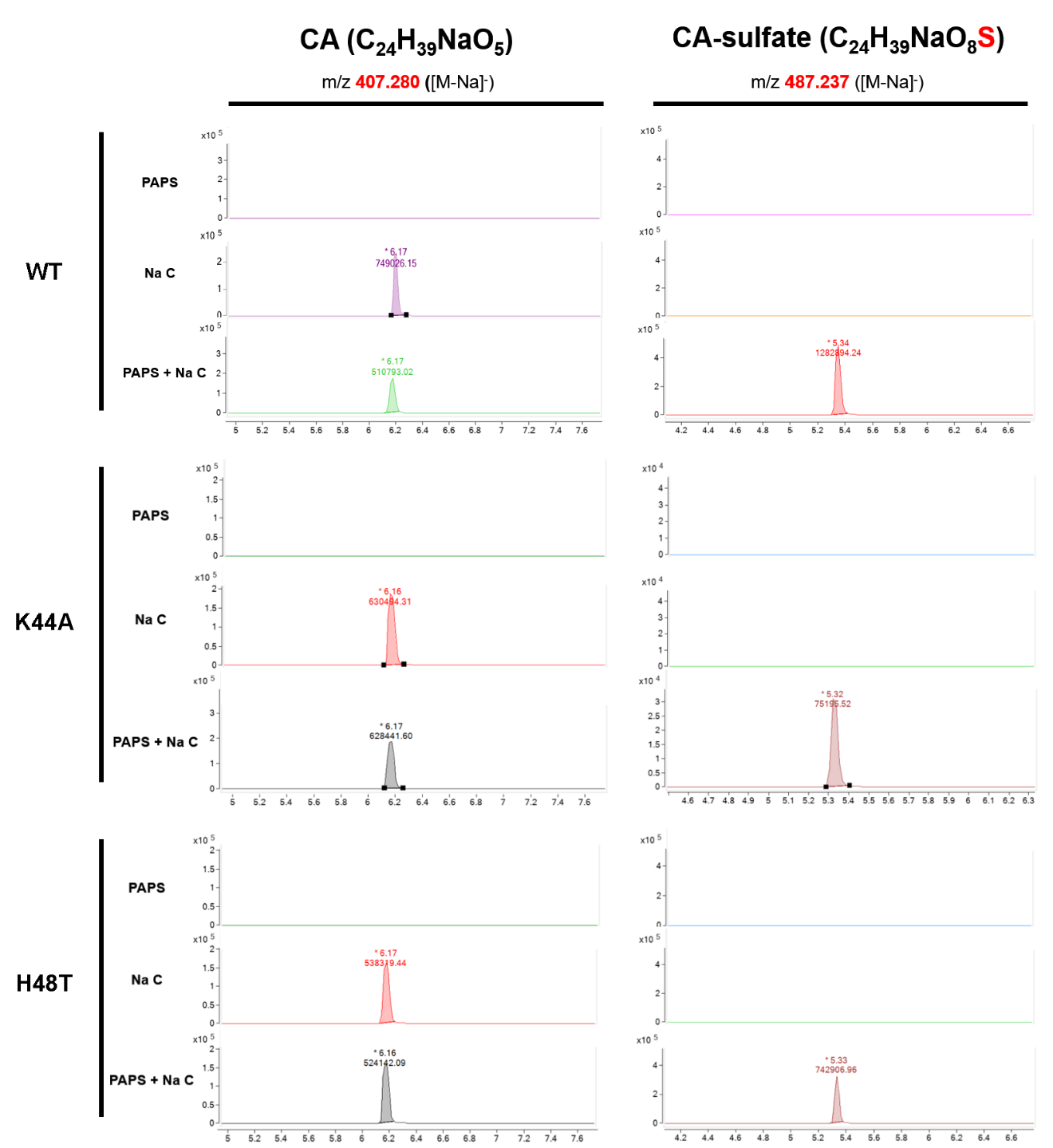


**Supplemental Fig. S3. Extracted-ion chromatograms of enzyme reaction mixtures of SULT2A8 WT, K44A and H48T.** The enzyme reactions of WT, K44A, H48T mutants toward Na C at pH 5.5 were set as described in Materials and Methods. The reaction mixtures were then subjected to LC-MS analysis to detect the conversion of CA (m/z, 407.280; retention, ~6.2 min) to CA-monosulfate (m/z, 487.237; retention, ~5.3 min). Retention times and peak areas were shown as indicated. PAPS only (PAPS) and Na C only (Na C) were used as controls. CA-monosulfate was produced and detected in reactions (PAPS + Na C) of both WT (peak area 1,282,894), K44A (peak area 75,196), and H48T (peak area 742,907).


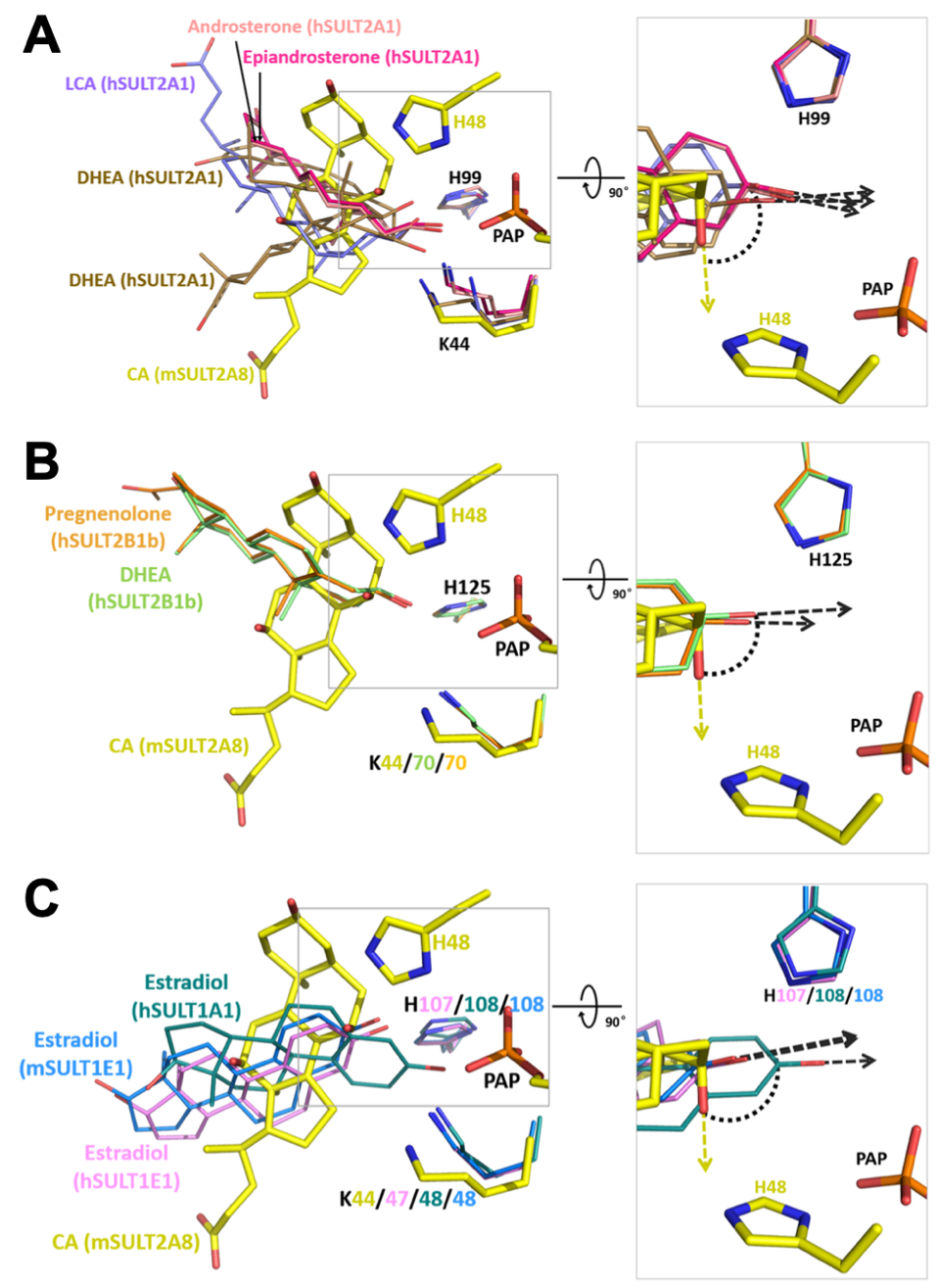


**Supplemental Fig. S4. Comparison of substrate orientation of mSULT2A8 with SULT1 and SULT2 isoforms.** Alignment of mSULT2A8 ligands and catalytic residues with hSULT2A1 models (A) (PDB ID 3F3Y, PAP+LCA; PDB ID 1J99, DHEA in two orientations; PDB ID 1OV4, androsterone; PDB ID 2QP3, epiandrosterone), hSULT2B1b models (B) (PDB ID 1Q20, PAP+pregnenolone; PDB ID 1Q22, PAP+DHEA), and SULT1 models complexed with estradiol (C) (hSULT1A1, PDB ID 2D06, PAP+estradiol; hSULT1E1, PDB ID 4JVL, PAP+estradiol; mSULT1E1, PDB ID 1AQU, PAP+estradiol). Direction of the target OH groups for sulfonation are indicated with dashed arrows.


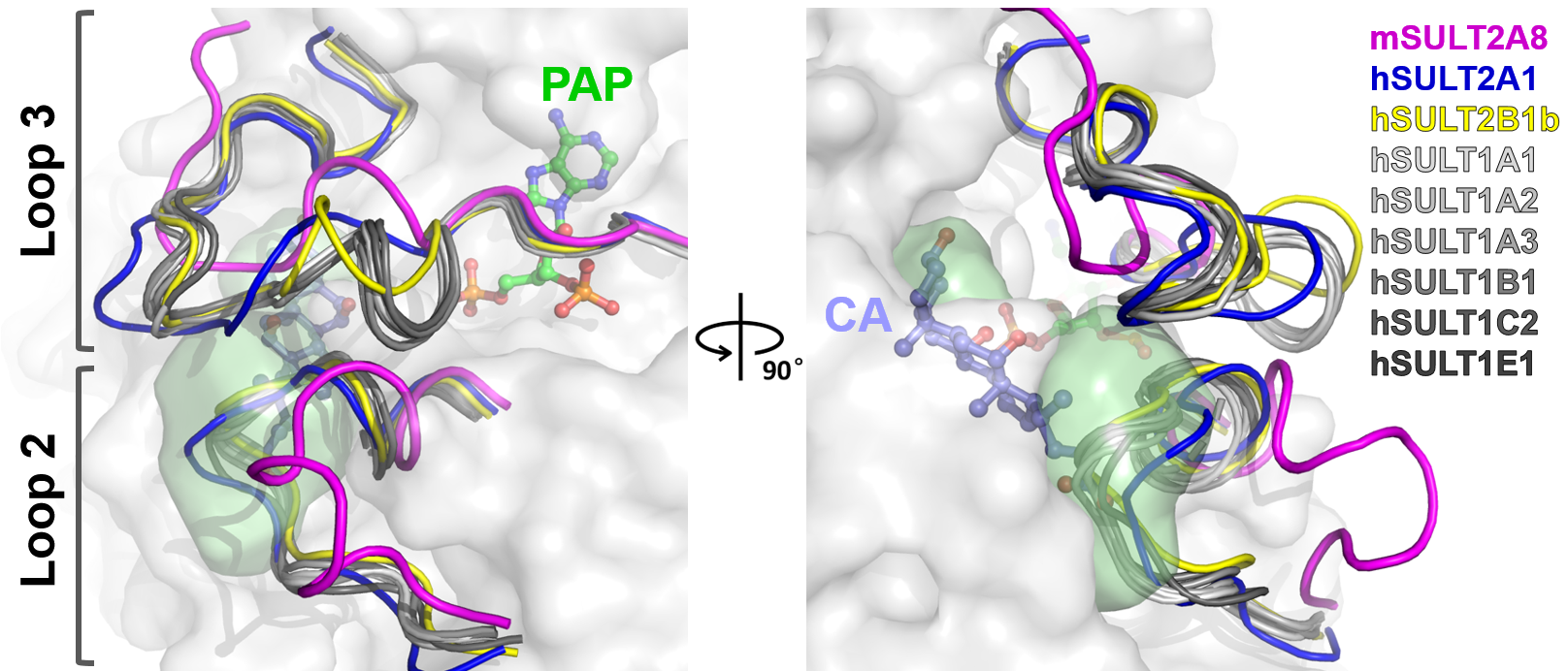


**Supplemental Fig. S5. Conformations of substrate binding loops in mSULT2A8.** Substrate binding loops 2 and 3 of mSULT2A8 were aligned with hSULT2A1 (PDB ID 3F3Y), hSULT2B1b (PDB ID 1Q20), hSULT1A1 (PDB ID 2D06), hSULT1A2 (PDB ID 1Z29), hSULT1A3 (PDB ID 2A3R), hSULT1B1 (PDB ID 3CK1), hSULT1C2 (PDB ID 2GWH) and hSULT1E1 (PDB ID 4JVL) structures. Molecular surface, bound ligands (CA and PAP) and substrate cavity (green) of mSULT2A8 structure are shown.


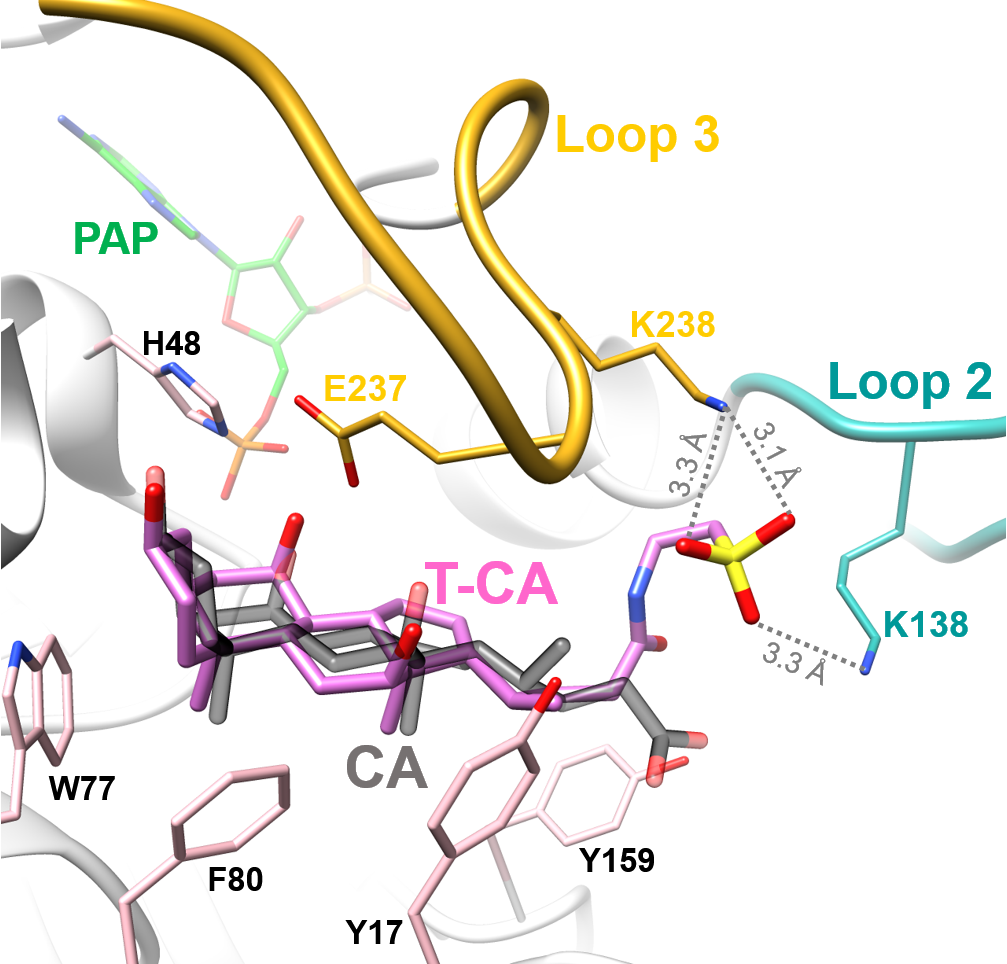


**Supplemental Fig. S6. Proposed interaction of T-CA with substrate binding loops 2 and 3.** T-CA (pink) was docked into the substrate binding pocket of SULT2A8 model using AutoDock Vina (74). CA bound in SULT2A8 is shown and colored in grey. Possible interactions and corresponding distances of Lys138 in loops 2 (green) and Lys238 in loop 3 (yellow) with the taurine end of the docked T-CA are indicated with dashed lines.
